# Repurposing of an old drug*: In vitro* and *in vivo* efficacies of buparvaquone against *Echinococcus multilocularis*

**DOI:** 10.1016/j.ijpddr.2018.10.011

**Published:** 2018-10-31

**Authors:** Reto Rufener, Luca Dick, Laura D'Ascoli, Dominic Ritler, Amani Hizem, Timothy N.C. Wells, Andrew Hemphill, Britta Lundström-Stadelmann

**Affiliations:** a Institute of Parasitology, Vetsuisse Faculty, University of Bern, Länggassstrasse 122, 3012, Bern, Switzerland; b Laboratory of Medical and Molecular Parasitology-Mycology, LR 12ES08, Department of Clinical Biology B, Faculty of Pharmacy of Monastir, University of Monastir, Monastir, 5000, Tunisia; c Medicines for Malaria Venture (MMV), Route de Pré-Bois 20, 1215, Geneva, Switzerland

**Keywords:** Pathogen box, MMV671636, MMV689480, Drug screening, Seahorse XFp analyzer, Cytochrome *bc*_1_ complex

## Abstract

The metacestode stage of the fox tapeworm *Echinococcus multilocularis* causes the lethal disease alveolar echinococcosis. Current chemotherapeutic treatment options are based on benzimidazoles (albendazole and mebendazole), which are insufficient and hence alternative drugs are needed. In this study, we screened the 400 compounds of the Medicines for Malaria Venture (MMV) Pathogen Box against *E. multilocularis* metacestodes. For the screen, we employed the phosphoglucose isomerase (PGI) assay which assesses drug-induced damage on metacestodes, and identified ten new compounds with activity against the parasite. The anti-theilerial drug MMV689480 (buparvaquone) and MMV671636 (ELQ-400) were the most promising compounds, with an IC_50_ of 2.87 μM and 0.02 μM respectively against *in vitro* cultured *E. multilocularis* metacestodes. Both drugs suggested a therapeutic window based on their cytotoxicity against mammalian cells. Transmission electron microscopy revealed that treatment with buparvaquone impaired parasite mitochondria early on and additional tests showed that buparvaquone had a reduced activity under anaerobic conditions. Furthermore, we established a system to assess mitochondrial respiration in isolated *E. multilocularis* cells in real time using the Seahorse XFp Analyzer and demonstrated inhibition of the cytochrome *bc*_1_ complex by buparvaquone. Mice with secondary alveolar echinococcosis were treated with buparvaquone (100 mg/kg per dose, three doses per week, four weeks of treatment), but the drug failed to reduce the parasite burden *in vivo*. Future studies will reveal whether improved formulations of buparvaquone could increase its effectivity.

## Highlights

• Screening 400 compounds against *E. multilocularis* metacestodes.
• Buparvaquone and ELQ-400 as two potent and novel drugs against *E. multilocularis*.
• Bioenergetics tests by Seahorse XFp analyzer in *E. multilocularis* germinal cells.
• Cytochrome *bc*_1_ complex is a molecular target of buparvaquone in *E. multilocularis*.
• Buparvaquone was not active against *E. multilocularis* in a murine model.

## Graphical abstract

The graphical abstract depicts *in vitro* cultured *E. multilocularis* metacestodes treated with 30 μM BPQ (on the right) for 5 days, a control-treated parasite specimen is shown on the right.

**Figure:**
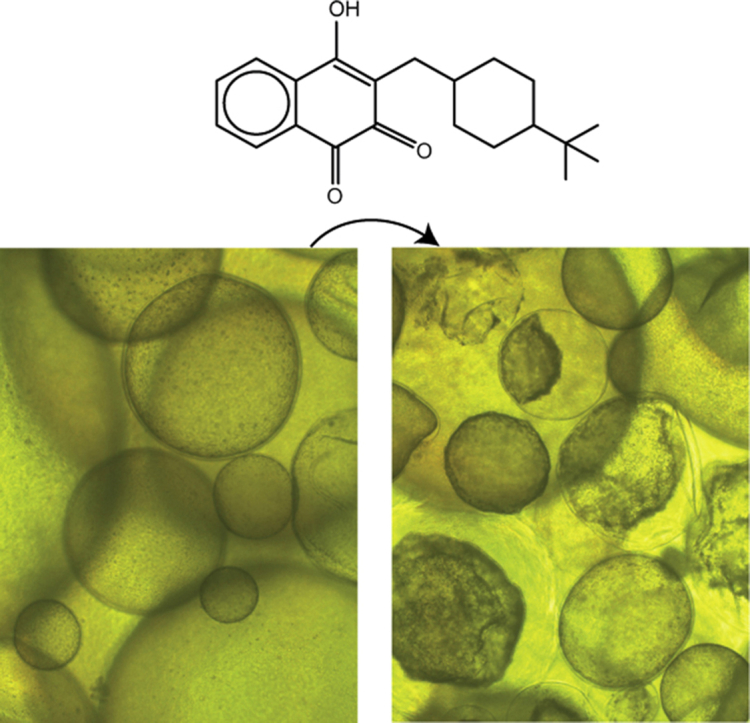

## Abbreviations

ABZ: albendazole
AE: alveolar echinococcosis
FBS: fetal bovine serum
EC_50_: half maximal effective concentration
ELQ: endochin-like quinolone
GL: germinal layer
HFF: human foreskin fibroblasts
LL: laminated layer
MAS: mitochondria assay solution
MIC: minimal inhibitory concentration
MMV: Medicines for Malaria Venture
OCR: oxygen consumption rate
PGI: phosphoglucose isomerase
RH: rat hepatoma
ROS: reactive oxygen species
TEM: transmission electron microscopy
TMPD: *N,N,N′,N′*-tetramethyl-*p*-phenylenediamine
Tx-100: Triton X-100

## 1 Introduction

Alveolar Echinococcosis (AE) is a life-threatening disease caused by infections with the fox tapeworm *Echinococcus multilocularis* which is endemic in the Northern hemisphere. The natural life cycle of *E. multilocularis* typically includes canids (often foxes) as definitive hosts and voles as intermediate hosts (Conraths and Deplazes, 2015). However, a large variety of mammals (including humans) can be infected as accidental intermediate hosts by ingesting parasite eggs shed by the definitive hosts during defecation. In humans, *E. multilocularis* forms larval metacestodes which primarily infect the liver, but they can also form metastases and affect other organs, especially at the late stage of infection (Kern, 2010). Metacestodes grow aggressively and infiltrate the host tissue, thus causing AE. AE has many pathological resemblances with a slow growing, malignant hepatic tumor, and for surgical excision of parasite lesions, the general rules of hepatic tumor surgery are followed accordingly (Kern et al., 2017). However, complete surgical removal of the parasitic lesions is often not possible, due to the diffuse and infiltrative nature of the metacestode tissue (Grüner et al., 2017; Kern et al., 2017). In such cases, chemotherapy remains the only widely used treatment option against AE. The current drugs of choice are the benzimidazole derivatives albendazole (ABZ) and mebendazole. However, they have several drawbacks, most importantly they act parasitostatic rather than parasiticidal (Hemphill et al., 2014, 2007), hence they have only limited potential to bring about a cure from infection, and massive doses of these drugs usually have to be administered throughout life (Kern et al., 2017). Additionally, benzimidazoles are not always well tolerated and can cause severe side effects, such as hepatotoxicity in some patients (Grüner et al., 2017). All these shortcomings make it urgent to develop alternative chemotherapeutic options against AE.

Given the relatively small target population, commercial support for neglected diseases such as echinococcosis is modest. Thus, one of the most promising strategies to find new drugs against AE (and likewise also other neglected diseases) is the repurposing of substances with already described activities against other pathogens. Open source drug discovery is fundamental to enable drug repurposing in an academic environment, and supported by organizations such as the Medicines for Malaria Venture (MMV) (Wells et al., 2016). MMV is a product development partnership with the declared goal of “[…] discovering, developing and facilitating the delivery of new, effective and affordable antimalarial drugs” (http://www.mmv.org). In 2013, MMV launched the open-access Malaria Box, a collection of 200 drug-like and 200 probe-like molecules with *in vitro* inhibitory activity against the malaria parasite *Plasmodium falciparum* (Spangenberg et al., 2013). The MMV Malaria Box was since then screened in over 290 assays against a wide range of organisms, including various parasites, bacteria, yeasts, and cancer cell lines (Voorhis et al., 2016). The 400 compounds from the Malaria Box were screened against *E. multilocularis* metacestodes, seven were found to be active *in vitro* at 1 μM, and one of them (MMV665807) was studied in more detail (Stadelmann et al., 2016). Following the success of the Malaria Box, MMV launched the Pathogen Box which contains 400 drug-like molecules with confirmed activity against various pathogens including parasites, bacteria, and viruses. Also included in the Pathogen Box are 26 reference compounds, which are well described drugs that are frequently used in clinical applications against various pathogens.

In this study, we screened the compounds from the MMV Pathogen Box *in vitro* against *E. multilocularis* metacestodes by applying the PGI-assay (as an indicator for physical drug-induced damage) and the Alamar Blue assay to monitor decreased viability of the metacestode tissue. Four compounds with promising activities were further tested for their cytotoxicity against rat hepatoma cells and human foreskin fibroblasts *in vitro*. Overall, we found two novel compounds with distinct activities against *E. multilocularis* metacestodes. One of them is buparvaquone (BPQ; MMV689480), which is a known anti-theilerial drug that was subsequently also tested in mice experimentally infected with *E. multilocularis*. To further study the mode of action of BPQ, we performed transmission electron microscopy (TEM) and established a system to measure its effect on the oxidative phosphorylation in the mitochondria of *E. multilocularis* cells.

## 2 Materials and methods

All chemicals were purchased from Sigma (St. Louis, MO, USA), unless stated otherwise. Dulbecco's modified Eagle medium (DMEM) and fetal bovine serum (FBS) were obtained from Biochrom (Berlin, Germany). The solutions containing Trypsin-EDTA, Penicillin/Streptomycin, and amphotericin B were purchased from Gibco-BRL (Zürich, Switzerland). The 400 compounds from the Pathogen Box were provided by MMV (Geneva, Switzerland) as 10 mM solutions in DMSO and stored at −20 °C. Additional samples of the compounds MMV021013, MMV671636, MMV687807 (provided by MMV), and BPQ (Cross Vet Pharm, Dublin, Ireland) were prepared as 10 mM stocks in DMSO upon arrival and stored at −20 °C.

### 2.1 *E. multilocularis* metacestode *in vitro* cultivation

*E. multilocularis* metacestodes were cultured as described by Spiliotis et al. (2004). In short, metacestodes (isolate H95) were grown *in vivo* in intraperitoneally (i.p.) infected Balb/c mice for 3–5 months. The parasite material was subsequently resected, pressed through a conventional tea strainer (Migros, Zürich, Switzerland), and incubated overnight at 4 °C in PBS containing 100 U/ml penicillin, 100 μg/ml streptomycin, and 10 μg/ml tetracycline. To establish a new *in vitro* culture, up to 2 ml of parasite tissue was co-cultured with 5 × 10^6^ Reuber rat hepatoma (RH) feeder cells and incubated at 37 °C with 5% CO_2_ in DMEM containing 10% FBS, 100 U/ml penicillin, 100 μg/ml streptomycin, and 5 μg/ml tetracycline. Once a week, the culture medium was changed and new RH cells were added. RH cells were cultured in parallel in the same culture medium, under the same conditions as the metacestodes, and they were passaged once a week.

### 2.2 Pathogen box screening design

The 400 compounds of the MMV Pathogen Box were initially screened at 10 μM in singlets by PGI-assay (see 2.3). The positive compounds from this initial screen were re-tested by PGI-assay in triplicates to confirm their activity at 10 μM. Thereafter, positive compounds were further tested at 1 μM in triplicates. Compounds were considered as active if they exceeded 20% PGI activity of the positive control Triton X-100 (Tx-100). After this screening cascade, four active compounds remained (BPQ, MMV021013, MMV671636, and MMV687807) that were serially diluted from 90 μM in 1:2 or 1:3 dilution steps to assess their EC_50_ values in triplicates. EC_50_ values were calculated after logit-log transformation in Microsoft Office Excel (2010). The three screening rounds of the Pathogen Box were each carried out once, and dilution series to assess the EC_50_ values were tested in three independent experiments. Mean values and standard deviations are given for the EC_50_ values.

### 2.3 *In vitro* drug testing of *E. multilocularis* metacestodes by PGI-assay

In order to assess the activity of compounds from the Pathogen Box on *E. multilocularis* metacestodes, the PGI-assay was employed. The PGI-assay measures the amount of the enzyme phosphoglucose isomerase (PGI) that metacestode vesicles release into the medium supernatant when their integrity is disrupted (Stadelmann et al., 2010). Metacestodes used for the PGI-assay were cultured *in vitro* for 4–10 weeks (diameter of 2–5 mm), washed in PBS, and mixed 1:1 with DMEM (supplemented with 100 U/ml penicillin, and 100 μg/ml streptomycin) before distribution in 48-well plates (1 ml vesicle suspension per well). Drugs were pre-diluted in DMSO and then added to the wells (1 μl per well). Corresponding amounts of DMSO were used as the negative control, and the nonionic surfactant Tx-100 (0.1% final concentration) was applied as positive control. The parasite- and drug-containing plates were incubated at 37 °C and 5% CO_2_, under humid atmosphere. To assess drug-induced metacestode damage by PGI-assay, 120 μl medium supernatant was collected from each well after 5 and 12 days of incubation and stored at −20 °C until further measurements were performed. The amount of PGI released in these media was measured as described by Stadelmann et al. (2010). The activity of PGI was finally calculated from the linear regression of the enzyme reaction over time and expressed as relative activity of the positive control Tx-100 in Microsoft Excel (2010) and Figures were prepared in Adobe Illustrator (2015).1.0.

### 2.4 Vesicle viability assay by Alamar Blue assay

After initial screening by PGI-assay, the vesicle viability assay by Alamar Blue was applied to the most active drugs (BPQ, MMV021013, MMV671636, and MMV687807). The setup was the same as for PGI-assay EC_50_ calculations and it was performed in triplicates. After 12 days of treatment, viability of metacestodes was measured by Alamar Blue assay as previously described (Stadelmann et al., 2016). Data was used to calculate the minimal inhibitory concentrations (MICs) of these compounds on metacestodes. The MIC was defined as the lowest concentration of a drug with no significant difference in viability compared to the Tx-100 control, where all parasites were dead (*p* > 0.05 in a one-tailed Students t-test). MICs were tested in three independent experiments and mean values and standard deviations were calculated in Microsoft Office Excel (2010).

### 2.5 Cytotoxicity measurements in human fibroblasts and rat hepatoma cells

The *in vitro* toxicity of selected compounds was tested against confluent and pre-confluent human foreskin fibroblasts (HFF) as well as RH cells. HFF were kept in DMEM supplemented with 10% FBS, 100 U/ml penicillin, 100 μg/ml streptomycin, and 0.25 μg/ml amphotericin B at 37 °C and 5% CO_2_ in a humid atmosphere. To start the assay, HFF were seeded in 96-well plates (10,000 cells per well for confluent cells and 1000 cells per well for pre-confluent cells). The cells were incubated in 100 μl HFF cultivation medium at 37 °C and 5% CO_2_ to attach to the well and let grow for 4 h (pre-confluent HFF) or 22 h (confluent HFF) before the drugs were added. Drugs were serially diluted starting at 100 μM in 1:2 or 3:4 dilution steps and added to the cells. The final dilution series was adapted individually for each drug. The cells were subsequently incubated for 5 days at 37 °C and 5% CO_2_ in humid atmosphere. RH cells were treated the same way as the HFF, with the difference that 50,000 cells were seeded per well to obtain a confluent monolayer, and 5000 cells per well for pre-confluent wells. RH cells were incubated in DMEM containing 10% FBS, 100 U/ml penicillin, 100 μg/ml streptomycin, and 5 μg/ml tetracycline.

To measure the viability of the cells after treatment, the Alamar Blue assay was employed (Stadelmann et al., 2016). Therefore, the cells were washed three times in PBS and resazurin was added to 10 mg/l. The fluorescence at 595 nm was subsequently measured after 0 h and 2 h (or after 0 h and 5 h for preconfluent HFF) with an EnSpire 2300 plate reader (PerkinElmer Life Sciences, Schwerzenbach, Switzerland). IC_50_ values were calculated in Microsoft Excel (2010) after logit-log transformation of relative growth. Each drug concentration was executed in triplicates for one experiment, and averages and standard deviations of three independent experiments were calculated for each drug.

### 2.6 Transmission electron microscopy

The preparation of the samples for transmission electron microscopy (TEM) was done according to the protocol of Hemphill and Croft (1997). In short, *E. multilocularis* metacestodes were distributed to 48-well-plates and incubated with DMSO or BPQ (30–0.04 μM) as described above. After an incubation period of 5 days, metacestodes were fixed in 2% glutaraldehyde in 0.1 M sodium cacodylate buffer; pH = 7.3 for 1 h. Next, the samples were stained for 2 h in a 2% osmium tetroxide solution cacodylate buffer, and subsequently pre-stained in a saturated uranyl acetate solution for 30 min. After washing the samples with water, they were dehydrated stepwise by washing in increasing concentrations of ethanol (30%, 50%, 70%, 90%, and three times 100%). The samples were then embedded in Epon 812 resin with three subsequent resin changes during 2 days and incubated at 65 °C overnight for polymerization. Sections for TEM (80 nm) were cut using an ultramicrotome (Reichert and Jung, Vienna, Austria), and were loaded onto formvar-carbon coated nickel grids (Plano GmbH, Marburg, Germany). The specimens were finally stained with uranyl acetate and lead citrate, and were viewed on a CM12 transmission electron microscope (Philips Electron Optics, Eindhoven, Netherlands) that operates at 80 kV.

### 2.7 Treatment of *E. multilocularis* metacestodes with BPQ under anaerobic/aerobic conditions

In an additional experiment, effect of oxygen on the activity of BPQ on *E. multilocularis* metacestodes was assessed. BPQ was serially diluted from 30 μM down to 4.57 nM in 1:3 dilution steps and added to metacestodes as described above. Corresponding DMSO controls were included. The plates with the metacestodes were incubated for 5 days either under aerobic conditions in a standard incubator (37 °C, supplemented with 5% CO_2_, humid atmosphere) or under anaerobic conditions at 37 °C in a defined gas mixture containing 80% N_2_, 10% CO_2_, and 10% H_2_, humid atmosphere. Subsequently, samples were taken for PGI-assay and processed as described above (2.3). The experiment was repeated three times independently. Figures were prepared in Adobe Illustrator (2015) 1.0.

### 2.8 Isolation of *E. multilocularis* germinal layer cells

To obtain germinal layer (GL) cells from *in vitro* grown metacestode vesicles, the protocol described by Spiliotis and Brehm (2009) was followed with few modifications. Prior to the isolation process, conditioned medium (cDMEM) was prepared as follows: 10^6^ RH cells were seeded in 50 ml DMEM (supplemented with 10% FBS, 100 U/ml penicillin, 100 μg/ml streptomycin, and 5 μg/ml tetracycline) in a T175 cell cultivation flask. These cells were incubated for 6 days at 37 °C with 5% CO_2_, under humid atmosphere. In addition, 10^7^ RH cells were cultivated the same way but incubated only for 4 days. After the incubation periods, medium supernatants were sterile filtrated, mixed 1:1, and stored at 4 °C until further use. To isolate GL cells, *E. multilocularis* metacestode vesicles (approximately 3 months old) from *in vitro* culture were harvested and washed in PBS. The vesicles were mechanically disrupted using a pipette. The remaining vesicle tissue was incubated in EDTA-Trypsin and occasionally gently shaken for 20 min. Thereafter the mixture was sieved through a 50 μm polyester tissue sieve (Sefar AG, Heiden, Switzerland) and rinsed with PBS. The flow-through containing the GL cells was collected, centrifuged, and the pellet was taken up in cDMEM. To standardize the amount of cells present in the mixture, the O. D. 600 of the cell suspension (diluted 1:100) was measured. An O. D. 600 of 100 was defined as one arbitrary unit per μl of the undiluted cell suspension. 700 units of GL cells were then seeded in 5 ml cDMEM and incubated overnight at 37 °C in a humified, oxygen-free environment of N_2_.

### 2.9 Assessment of mitochondrial respiration in *E. multilocularis* GL cells by Seahorse XFp analyzer

A Seahorse XFp Analyzer (Agilent Technologies, Bucher Biotec, Basel, Switzerland) was used to assess the oxygen consumption rate (OCR) as an indicator of the mitochondrial respiration of *E. multilocularis* GL cells in real time. Plasma membrane permeabilizer (PMP, Agilent Technologies), was applied to selectively permeabilize only the plasma membrane of GL cells and thereby exposing the mitochondria directly to the assay medium. The assays were done according to the manufacturer's manuals and to Divakaruni et al. (2014).

One day prior to the assay, the sensor cartridge was hydrated overnight in XF calibrant solution (Agilent Technologies) at 37 °C and a Seahorse XFp miniplate was coated with CellTak (Fisher Scientific, Schwerte, Germany) according to the manufacturer's protocol to prepare them for cell attachment.

The assays were carried out in mitochondria assay solution (MAS) which consisted of 220 mM mannitol, 70 mM sucrose, 10 mM KH_2_PO_4_, 5 mM MgCl_2_, 2 mM HEPES, and 1 mM EGTA, at a pH of 2.7. A stock solution of 3 x MAS was prepared as described by the manufacturer's manual and stored at 4 °C, and BSA was added to 1 x MAS at a final concentration of 0.2% for each assay (assay medium). To run an assay, the test compounds to be injected were prepared as ten times stock in MAS and then loaded to the delivery ports of the sensor cartridge. The final concentrations of the test compounds for injections were 1 μM (BPQ), 10 mM (succinate and glycerol-3-phosphate), 20 mM (ascorbate) and 0.6 mM (*N,N,N′,N′*-tetramethyl-*p*-phenylenediamine (TMPD)). GL cells that had been isolated the previous day (section 2.8) were washed in MAS and taken up in assay buffer which consisted of 1x MAS supplemented with 10 mM succinate, 2 μM rotenone, 4 mM ADP, and 3.6 nM PMP. The cells were then distributed to a CellTak coated XFp miniplate with 50 units GL cells per well in 180 μl assay medium. The plate was centrifuged at 300 g for 1 min and transferred to the Seahorse XFp Analyzer to start measurements with 30 s mix time, 30 s delay time, and 2 min measure time without an equilibration step. BPQ was injected after the fourth measurement, and after the seventh measurement the substrates of interest (succinate, glycerol-3-phosphate, or ascorbate/TMPD) were added to the wells. Measurements were performed in triplicates and data analysis was performed in Wave (version 2.6, Agilent Technologies). The experiment was repeated three times, and one representative figure is shown. The figure was prepared in Adobe Illustrator (2015).1.0.

### 2.10 Ethic statements and animal maintenance

The *in vivo* studies were performed in compliance with the Swiss animal protection law (TschV, SR 455). The study was approved by the Animal Welfare Committee of the Canton of Bern (license number BE 112/14).

Balb/c mice, 6 weeks old, were purchased from Charles River Laboratories (Sulzheim, Germany) and used for *in vivo* experiments when they were 8 weeks old and weighted approximately 20 g. The mice were housed in a type 3 cage containing enrichment in the form of a cardboard house and paper and woodchip bedding with a maximum of seven mice per cage. They were maintained in a 12 h light/dark cycle, controlled temperature of 21 °C–23 °C, and a relative air humidity of 45%–55%. Food and water was provided *ad libitum*.

### 2.11 BPQ treatment of *E. multilocularis* infected mice

Experimentally infected mice were treated with BPQ to elucidate the efficacy of the drug *in vivo.* To infect mice, *in vitro* grown *E. multilocularis* metacestodes (isolate H95) were washed in PBS, were mechanically destroyed by pipetting and the resulting suspension was centrifuged for 5 min at 500 g. The parasite tissue was then taken up in an equal volume of PBS. Each mouse was subsequently infected intraperitoneally (i.p.) with 200 μl of this suspension. 32 infected mice were randomly distributed into 3 treatment groups (8 animals per group) with 4 animals per cage. Group 1 (negative control) received only the solvent corn oil; group 2 (positive control) received ABZ (200 mg/kg per day); and group 3 received BPQ (100 mg/kg per day). Treatments of mice started 2 weeks post-infection and lasted for 4 weeks, with consecutive treatment of mice for 5 days per week, followed by an interruption of treatment for 2 days for recovery. All treatments were administered by oral gavage in a volume of 50 μl, with ABZ and BPQ being suspended in corn oil. After four weeks, all mice were anesthetized with isoflurane and subsequently euthanized by CO_2_. The parasitic tissue from each mouse was completely resected and weighed. The mass of the resected parasitic tissue was used for statistical analyses of the experiment. The three groups were analyzed by two-sided exact Wilcoxon rank-sum test and *p*-values were Bonferroni adjusted (R version 3.4.2). The significance level was set to *p* < 0.05. Figures were prepared in R and Adobe Illustrator 2015.1.0.

## 3 Results

### 3.1 Screening the Pathogen Box identifies four compounds with promising *in vitro* activity against *E. multilocularis* metacestodes

**Fig. 1:**
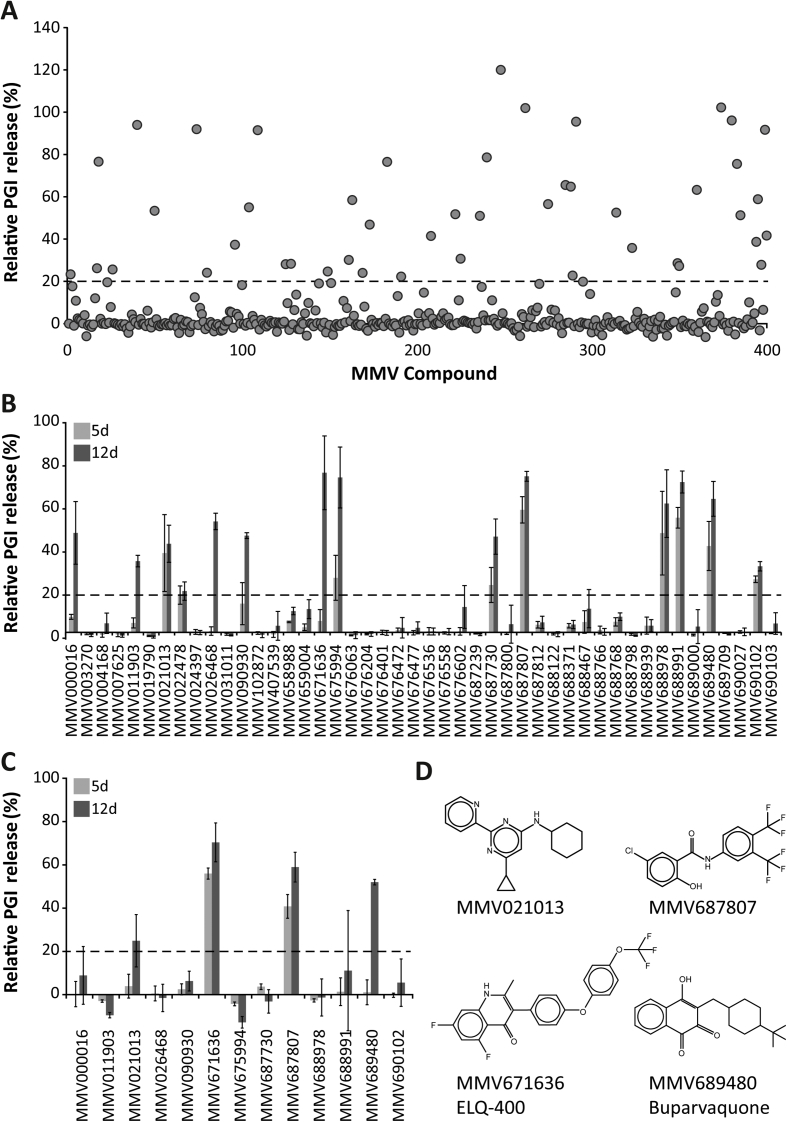
**Screening of the MMV Pathogen Box on *E. multilocularis* metacestodes *in vitro*.** (A–C) Relative PGI release as assessed by PGI-assay is shown. 100% PGI release was defined as the release upon treatment with the positive control Tx-100 (0.1%). Compounds were considered as active if they exceeded 20% relative PGI release (dashed line). (A) Initial screen of the 400 compounds of the MMV Pathogen box at 10 μM in singlets after 12 days of treatment (values for 5 and 12 days are given in Supplementary Table 1). MMV compounds are listed in numerical sequence. (B) Confirmation of active compounds by testing at 10 μM in triplicates (values are given in Supplementary Table 2). (C) Testing of active compounds from (B) at 1 μM in triplicates (values are given in Supplementary Table 3). PGI release is shown for 5 and 12 days of treatment in (B) and (C). Data is represented as means and standard deviations. (D) Structural formula of the four compounds that were active at 1 μM and further followed.

400 compounds from the MMV Pathogen Box were initially screened *in vitro* on *E. multilocularis* metacestodes at 10 μM. This screen was carried out in singlets and resulted in 13 active compounds after 5 days and 46 active compounds after 12 days of incubation (Fig. 1A). The 46 compounds that were positive in the initial screen were confirmed in a second screening round at 10 μM in triplicates to exclude false-positives. This yielded 8 positive hits after 5 days, and 5 additional active compounds after 12 days (13 active compounds in total; Fig. 1B). From these active compounds, four were reference compounds of the Pathogen Box (MMV000016, mefloquine; MMV688978, auranofin; MMV688991, nitazoxanide, and MMV689480, BPQ), four compounds were from the tuberculosis disease set (MMV021013, MMV090930, MMV687730, and MMV687807), two compounds were from the malaria disease set (MMV011903 and MMV026468), and one compound each was from the onchocerciasis, cryptosporidiosis, and kinetoplastid disease set (MMV671636, MMV675994, and MMV690102). In order to assess the efficacies of those 13 active compounds at low concentrations, they were further tested at 1 μM in triplicates (Fig. 1C). Four compounds (BPQ, MMV021013, MMV671636, and MMV687807, see Fig. 1D) were found to exhibit distinct *in vitro* activities against metacestodes at this lower concentration. The numerical results of the full screening of the compounds from the Pathogen Box are provided in Supplementary Tables 1-3. Subsequently, we assessed EC_50_ and MIC values on *E. multilocularis* metacestodes for these four compounds (Table 1). The EC_50_ are representative for the activity in the metacestode PGI-assay, and the MIC for the parasiticidal potential in the vesicle viability assay by Alamar Blue assay. The compound with the highest activity after 5 and 12 days of incubation was MMV671363, followed by MMV687807, both with 5 day EC_50_ and MIC values below 1 μM. BPQ was a little less active after 5 days, but activity also increased to the sub-micromolar range at day 12. MMV021013 did not exhibit a specifically low EC_50_, although longer exposure of metacestodes to the drug increased its efficacy as well.

**Table 1:** **Summarized parasite toxicity and mammalian cell toxicity of the four most active compounds from the MMV Pathogen box.** EC_50_ and IC_50_ values were calculated based on PGI-assay (for *E. multilocularis* metacestodes) and Alamar Blue assay (for mammalian cells) after incubation for 5 days *in vitro*. MIC values were calculated based on Alamar Blue vesicle viability assay after 12 days of incubation. Mean values and standard deviations (in parentheses) from at least three independent experiments are given in μM.

	BPQ; MMV689480		MMV021013		MMV671636		MMV687807	
*E. multilocularis* EC_50_	2.87	(1.45)	15.75	(8.75)	0.02	(0.01)	0.36	(0.16)
*E. multilocularis* MIC	0.56	(0.33)	20	(10.00)	0.01	(0.00)	0.59	(0.44)
HFF preconfluent IC_50_	5.99	(3.39)	5.14	(2.74)	12.31	(6.95)	0.15	(0.02)
HFF confluent IC_50_	48.61	(8.83)	53.09	(5.1)	74.82	(38.43)	0.37	(0.07)
RH preconfluent IC_50_	8.47	(3.89)	1.1	(0.77)	0.25	(0.11)	1.27	(0.23)
RH confluent IC_50_	17.42	(2.09)	2.07	(0.35)	2.07	(1.67)	20.13	(10.92)

### 3.2 Cytotoxicity measurements on pre-confluent and confluent HFF and RH cell cultures identifies two compounds with specific activity against *E. multilocularis* metacestodes

We determined the IC_50_ values of BPQ, MMV021013, MMV671636, and MMV687807 on mammalian RH cells and HFF (Table 1). Large differences between the IC_50_ values were observed depending on the confluence and type of the host cell, but all four compounds had commonly lower IC_50_ values against pre-confluent cells than against confluent cells. BPQ was less toxic against all tested host cells than against *E. multilocularis* metacestodes. MMV021013 was generally as toxic to host cells as it was to *E. multilocularis* metacestodes; only confluent HFF were more resistant. MMV671363 was less toxic against all tested cell lines than against *E. multilocularis*, indicating a potential therapeutic window. Additionally, it had a notably lower IC_50_ for RH cells than for HFF. MMV687807 showed the highest toxicity against HFF, and accordingly this compound could only exhibit a potential therapeutic window for RH cells. Taken together, only BPQ and MMV671363 exhibited specific toxicity against *E. multilocularis* metacestodes.

Since BPQ is an already marketed drug for the treatment of theileriosis in cattle, and other potential applications include leishmaniasis and babesiosis, this compound was chosen for further characterization.

### 3.3 Transmission electron microscopy of BPQ-treated metacestodes reveals distinct changes in the mitochondrial ultrastructure

**Fig. 2:**
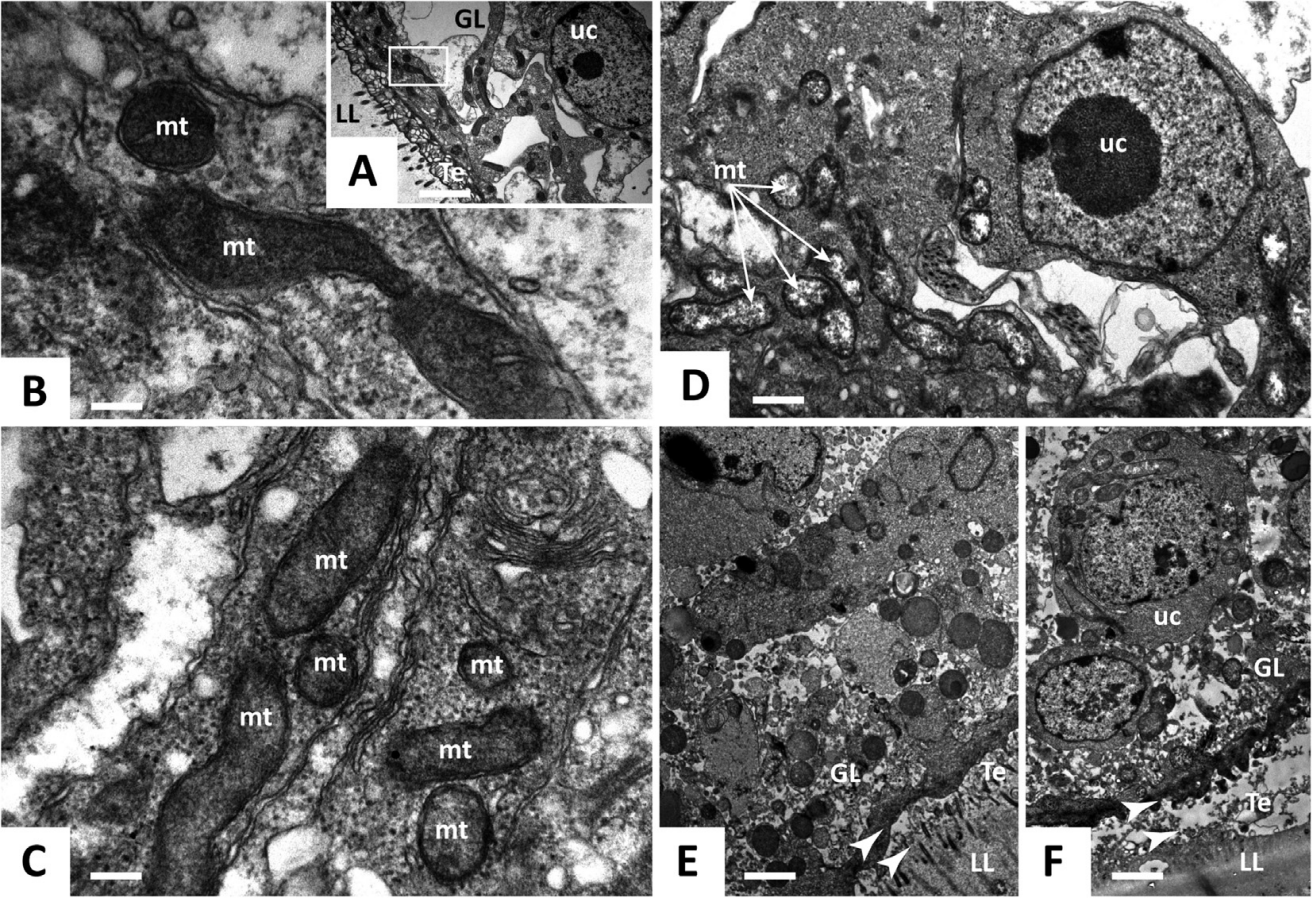
**Transmission electron microscopy of *E. multilocularis* metacestodes treated by BPQ *in vitro*.** (A–C) Control-incubated metacestodes with mitochondria. (D) Metacestodes treated with 0.3 μM BPQ showing first signs of altered mitochondria (indicated by arrows). (E) Metacestodes treated with 1 μM BPQ. Note the partial separation of the laminated layer from the tegument and the membrane stacks (indicated by arrow heads). (F) Metacestodes treated with 3 μM BPQ. The laminated layer is completely detached from the tegument, as indicated by arrow heads. Abbreviations: GL, germinal layer; LL, laminated layer; Te, tegument; mt mitochondria; uc undifferentiated cell. Thin arrows depict mitochondria and arrow heads show the separation of the laminated layer from the tegument. The respective size bars are in A = 2.8 μm, in B = 0.26 μm, in C = 0.32 μm, in D = 1 μm, in E = 2.2 μm, and in F = 2.2 μm.

The morphological alterations induced by BPQ on *E. multilocularis* metacestodes were thoroughly investigated by TEM (Fig. 2). The *E. multilocularis* metacestode is composed of two layers: an outer, acellular and protective layer (the laminated layer, LL) that is composed of highly glycosylated mucins, and an inner layer denominated GL, where various cells (including muscle cells, lipid storage cells, nerve cells, and undifferentiated stem cells) reside. In between the LL and the GL is the tegument, which is a syncytial tissue containing villi-like microtriches that protrude into the LL. *In vitro*-cultured *E. multilocularis* metacestodes were cultured in the presence of different concentrations of BPQ during 5 days. Ultrastructural damage was observed at concentrations as low as 0.3 μM (Fig. 2D): The most distinct effects at this low concentration were seen within the mitochondria, which appeared less electron dense than those of the untreated control. At 1 μM, membrane stacks were observed, and the GL started to separate from the LL (Fig. 2E). The metacestode integrity was seriously impaired at 3 μM of BPQ, with the LL being detached completely from the GL (Fig. 2F). Due to the alterations of mitochondria upon treatment with BPQ, further studies on the mode of action of BPQ in *E. multilocularis* focused on oxygen-dependence.

### 3.4 Under anaerobic culture conditions, the activity of BPQ against *E. multilocularis* metacestodes is dramatically diminished

**Fig. 3:**
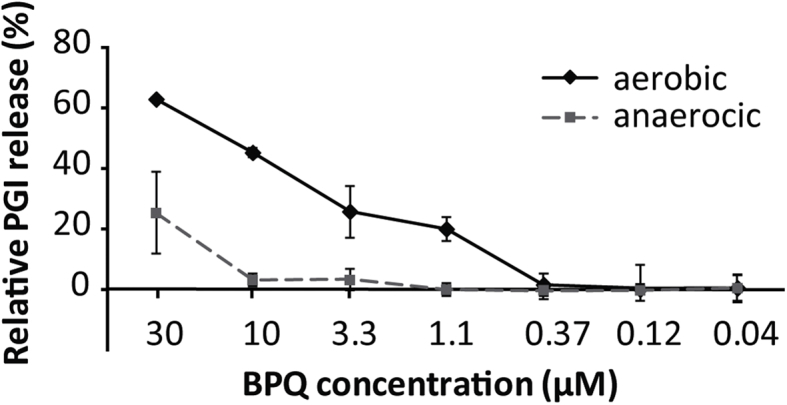
***In vitro* activity of BPQ on *E. multilocularis* metacestodes under anaerobic conditions.** Relative PGI release as assessed by PGI-assay is shown. 100% PGI release was defined as the release upon treatment with Tx-100 (0.1%) *E. multilocularis* metacestodes were incubated for 5 days in the presence of various concentrations of BPQ (30–0.04 μM) under aerobic versus anaerobic conditions. The experiment was performed three times and in triplicates. Mean values and standard deviations from one representative experiment are shown.

As assessed by PGI-assay, incubation of *E. multilocularis* metacestodes under anaerobic conditions resulted in a reduction of activity of BPQ. After 5 days of incubation in an oxygen-free atmosphere, the drug did not induce damage on metacestodes at 10 μM or lower concentrations. Only at 30 μM BPQ was active, and less pronounced compared to metacestodes that were incubated under aerobic conditions (Fig. 3).

### 3.5 BPQ treatment inhibits mitochondrial respiration in *E. multilocularis* GL cells

**Fig. 4:**
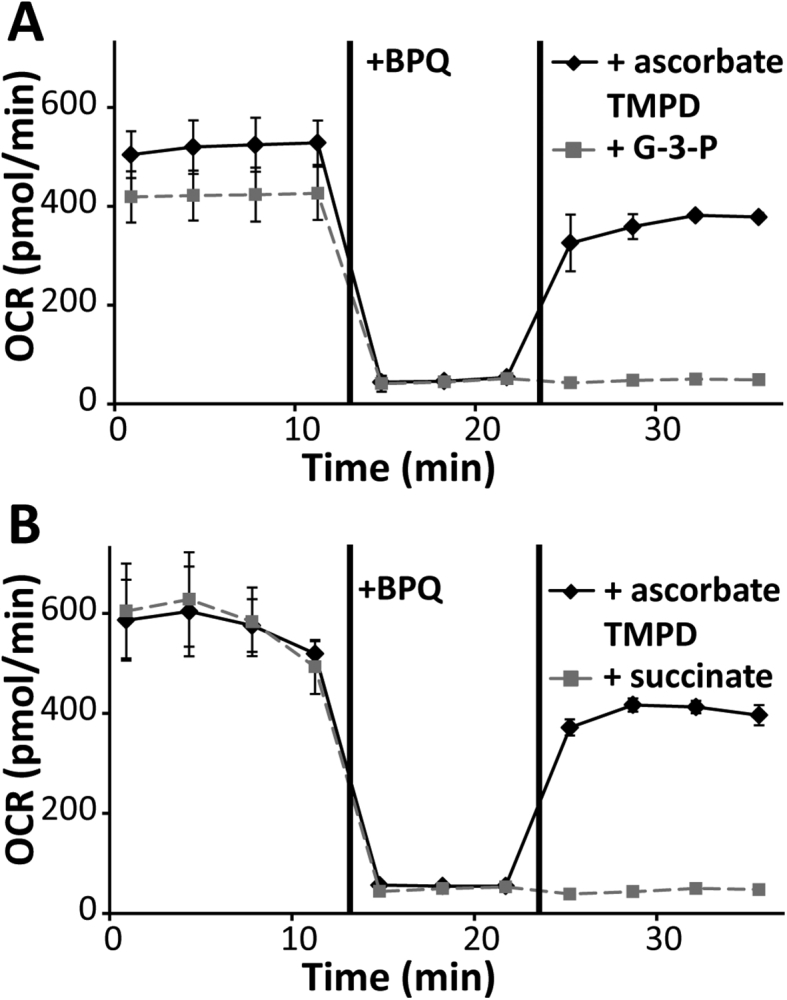
**Mitochondrial respiration in *E. multilocularis* GL cells.** The oxygen consumption rate (OCR) of isolated, permeabilized GL cells of *E. multilocularis* was assessed using as Seahorse XFp Analyzer. The cells were initially fed with succinate as an electron donor. The OCR dropped after the cells were exposed to 1 μM BPQ, but recovered again after the addition of ascorbate (20 mM) with TMPD (0.6 mM), which donate electrons to mitochondrial respiration chain complex IV. The addition of 10 mM glycerol-3-phosphate (G-3-P, A) or 10 mM succinate (B) had no effect on the OCR after BPQ treatment. The experiment was repeated three times and one representative plot is shown. Measurements were done in triplicates, and values are given as means with standard deviations.

To further elucidate the mode of action of BPQ, we established an *in vitro* system using a Seahorse XFp analyzer and isolated, permeabilized GL cells of *E. multilocularis* that allows us to monitor the mitochondrial respiration. The Seahorse XFp analyzer measures the OCR of cells, which directly correlates with the activity of mitochondrial complex IV. After addition of 1 μM BPQ to *E. multilocularis* GL cells, the OCR rapidly decreased (Fig. 4). Moreover, addition of ascorbate together with TMPD could restore the OCR (Fig. 4), and ascorbate/TMPD are generally known to feed electrons directly into complex IV. However, neither the addition of succinate (the substrate of complex II of the mitochondrial respiratory chain), nor glycerol 3-phosphate (which donates electrons to coenzyme Q via mitochondrial glycerol 3-phosphate dehydrogenase), could restore the OCR, as they are both taken up upstream of complex III. Taken together, this strongly suggests that BPQ selectively inhibits complex III in the mitochondrial electron transport chain of *E. multilocularis* GL cells.

### 3.6 BPQ treatment of infected mice does not result in a reduction of parasite burden

**Fig. 5:**
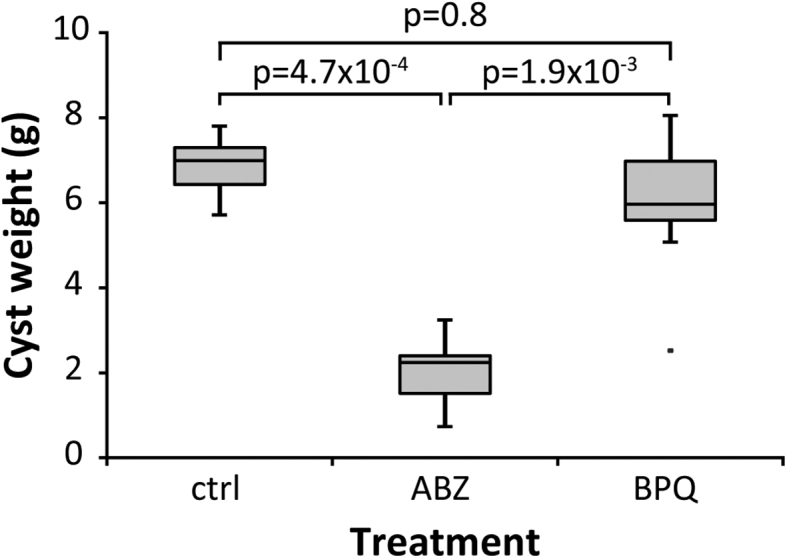
***In vivo* treatment of *E. multilocularis* infected mice with BPQ.** Balb/c mice were intraperitoneally infected with *E. multilocularis* metacestodes 2 weeks prior to drug treatment. Compounds were administered by p.o. gavage in a volume of 50 μl in corn oil. ABZ (200 mg/kg, n = 8), BPQ (100 mg/kg, n = 8), and corn oil only (ctrl, n = 8) were administered five times per week. After four weeks of treatment, mice were sacrificed, and parasite cysts were resected and weighted. The data is represented as box-whisker plots.

The *in vivo* efficacy of BPQ treatment was assessed in experimentally infected Balb/c mice. Mice were treated during 4 weeks p.o. with 100 mg/kg BPQ during 5 days per week. ABZ (200 mg/kg during 5 days per week as the standard drug for patients suffering from AE was used as a positive control (Fig. 5). None of the mice showed signs of adverse effects due to treatment with BPQ or ABZ during the whole course of treatment. While treatment with ABZ led to a significant reduction in parasite burden when compared to the control (Bonferroni adjusted *p*-value = 4.7*10^−4^) or the BPQ treated group (Bonferroni adjusted *p*-value = 1.9 * 10^−3^), there was no significant difference between the control group and the BPQ treated group (Bonferroni adjusted *p*-value = 0.8; Fig. 5).

## 4 Discussion

Alveolar echinococcosis (AE) is a serious and life-threatening disease caused by the cestode *E. multilocularis*. Current chemotherapies rely on benzimidazole treatment. However, they are insufficient since they can cause severe side effects, and they can only inhibit the growth and dispersion of metacestodes, but do not kill the parasite (Hemphill et al., 2014). Thus, alternative treatment options are urgently needed.

In recent years, major advances have been achieved for the *E. multilocularis* model. These include the development of new *in vitro* culture methods which allow the large-scale production of metacestode vesicles (Spiliotis and Brehm, 2009), as well as the introduction of the PGI-assay as a medium-throughput drug-screening method providing an objective read-out (Stadelmann et al., 2010). These breakthroughs enabled the screening of hundreds of compounds against *E. multilocularis*. An *in vitro* cascade to screen drug libraries against *E. multilocularis* has recently been introduced by Stadelmann et al. (2016) and it was applied to the MMV Malaria Box.

In the present study, we screened the MMV Pathogen Box *in vitro* for active compounds against *E. multilocularis* metacestodes. From the 400 compounds, 13 (or 3.25%) were active at 10 μM and 4 (or 1%) of these also at 1 μM. This is a similar hit ratio when compared to the outcome of the MMV Malaria box, where 24 (6%) and 7 compounds (1.75%) were found to be active at 10 μM and 1 μM respectively (Stadelmann et al., 2016). Of the four compounds that were active at 1 μM, only BPQ and MMV671636 exhibited a high specificity against the parasite. MMV021013 showed only a moderate EC_50_ against *E. multilocularis* metacestodes and was as toxic to mammalian cells as it was against the parasite. MMV687807 was very effective against *E. multilocularis*, but unfortunately also exhibited substantial toxicity against HFF. Interestingly, MMV687807 is structurally very similar to MMV665807, the top hit from the screening of the Malaria Box against *E. multilocularis* (Stadelmann et al., 2016). However, MMV665807 did not exhibit any specific toxicity against HFF, in contrast to the here tested MMV687807. Both, MMV665807 and MMV687807, are salicylanilide-derivatives related to the well-known anthelmintic niclosamide, with the only difference that MMV687807 has an additional trifluoromethyl group attached to the benzene ring. Both BPQ and MMV671636 were highly active against *E. multilocularis* metacestodes and less against mammalian cells, thus suggesting for a potential therapeutic window and rendering these two compounds suitable for further analyses. MMV671636 (also known as ELQ-400) belongs to a group of novel anti-malarial compounds called endochin-like quinolones (ELQ), some of which, including ELQ-400, also exhibit excellent activities against other apicomplexan parasites such as *Toxoplasma*, *Babesia* and *Neospora* (Lawres et al., 2016; Müller et al., 2017). We here further focused on the marketed hydroxynaphthoquinone BPQ, which is related to parvaquone and ubiquinone and currently used in the treatment of theileriosis in cattle. BPQ also has reported *in vivo* activity against *Leishmania* spp. in mice and *Babesia equi* in horses (Croft et al., 1992; Zaugg and Lane, 1992). It has been shown that BPQ acts via a mechanism involving the inhibition of cytochrome *bc*_1_ complex in the mitochondria of *Theileria* (Ortiz et al., 2016). Another study in *Theileria annulata* suggested that BPQ is also targeting the peptidyl-prolyl isomerase PIN1 (Marsolier et al., 2015). According to our TEM observations, the mitochondria of *E. multilocularis* metacestodes are among the first structures to be affected when treated with BPQ. Moreover, we confirmed the mitochondrial cytochrome *bc*_1_ complex (complex III) as a molecular target of BPQ in *E. multilocularis*. The Seahorse technology that was applied to perform these experiments has already been employed to study the metabolism of the trematode *Schistosoma mansoni* (Huang et al., 2012), the nematodes *Caenorhabditis elegans* and *Haemonchus contortus* (Luz et al., 2015; Preston et al., 2016), but so far never for any cestode or isolated helminth cells.

The cytochrome *bc*_1_ complex has already before proven its value as a valid antiparasitic drug target: Atovaquone for example is another hydroxynaphthoquinone (like BPQ) and a potent inhibitor of the cytochrome *bc*_1_ complex. It is currently widely used (in combination with proguanil) to treat and prevent malaria, especially in chloroquine resistant patients (Birth et al., 2014).

In *E. multilocularis* metacestodes, the *in vitro* activity of BPQ decreased under anaerobic conditions. *E. multilocularis* can perform fermentation (lactic acid, ethanol) under anaerobic conditions (Agosin, 1968; McManus and Smyth, 1978). In addition, as for many other parasitic flatworms, *Echinococcus* can perform malate dismutation to ferment carbohydrates under anaerobic conditions, and is thus not totally dependent on the mitochondrial respiration chain (Tsai et al., 2013). This could explain, why BPQ is not highly active under anaerobic conditions. However, as for the *in vivo* situation, it is expected that the parasite is depending on a combination of aerobic and anaerobic energy generating pathways and that it encounters at least microaerobic conditions in the liver (Bryant, 1970). Our *in vivo* trial in experimentally infected mice demonstrated that there was no statistically significant reduction in parasite burden upon treating *E. multilocularis* infected mice p.o. with BPQ. One important reason for this discrepancy between *in vitro* and *in vivo* activity could be explained by the fact that *in vitro* screening was performed in the absence of any serum, as the assay was initially established without FBS due to interference with the test. Another reason for failure of the drug against murine AE could be the experimental model, which is based on artificial injection of parasite metacestodes into the peritoneal cavity of mice, and thus growth of parasites occurs primarily there. Upon natural infection of mice with *E. multilocularis* eggs, where the parasite grows primarily in the liver, higher oxygen concentrations might be reached, and thus also higher effectiveness of BPQ would be expected. A further explanation for the different outcome of *in vitro* and *in vivo* treatment of the parasite with BPQ could lay in its mode of action: Blocking the electron transport chain in the mitochondria is expected to lead to the generation of toxic reactive oxygen species (ROS) (Ortiz et al., 2016). Whereas the parasite *E. multilocularis* is known to be sensitive against ROS as it is lacking some of the key enzymes for ROS detoxification (e.g. catalase), *E. multilocularis* metacestodes might be better protected from ROS in an *in vivo* setting where detoxifying host cells are closely surrounding the parasite (Spiliotis and Brehm, 2004; Williams et al., 2013). However, the topic of ROS in echinococcosis awaits further investigation in the future. A third drawback of BPQ is its poor solubility and consequently poor bioavailability, and in particular poor entry into the parasitic tissue, which might be a further explanation for lack of *in vivo* efficacy thus far. Within the present study, neither plasma levels nor BPQ concentrations within the metacestodes were determined. Only one study so far measured BPQ levels in orally treated mice (Smith et al., 2018), and reached a C_max_ of 1.2 μM when treating with a single dose of 6 mg/kg. Assuming linear correlation, extrapolation of this dosage to the here applied 100 mg/kg would result in a C_max_ of 20 μM, which is above the EC_50_ of BPQ against *E. multilocularis* metacestodes *in vitro*. Some attempts to increase the bioavailability of BPQ were made in the past, such as formulation of better soluble oxime- and phosphate derivatives (Mäntylä et al., 2004), which show higher efficacies against leishmaniasis *in vivo* (Garnier et al., 2007). Solid lipid nanoparticles loaded with BPQ were also generated, but these nanoparticles were never tested against parasites (Soni et al., 2014). More recently, Smith and colleagues (Smith et al., 2018) presented a BPQ loaded self-nanoemulsifying drug delivery system, which showed a slightly increased bioavailability, compared to an aqueous dispersion of BPQ, after oral administration in mice. Such formulations of BPQ should be tested in the future also for their efficacy against AE in mice.

Several compounds from the MMV Pathogen Box were already tested before against *E. multilocularis* or *E. granulosus in vitro* and/or *in vivo*. Pentamidine (MMV000062), alpha-difluoromethylornithine (MMV001625), and suramine (MMV637953) were all tested *in vivo* against *E. granulosus*, but did not show any effects (Kammerer and Perez-Esandi, 1975; Miyaji et al., 1993). Rifampicin (MMV688775) and miltefosine (MMV688990) were both tested *in vitro* against *E. multilocularis* metacestodes (Reuter et al., 2006) and rifampicin was also tested *in vivo* (Kammerer and Perez-Esandi, 1975). However, both compounds were ineffective in these studies. In accordance to these findings, the compounds were also inactive in the present *in vitro* screen against *E. multilocularis*. Praziquantel (MMV002529), despite its wide use against intestinal infections with adult cestodes (including *Echinococcus* spp.) and other parasites, is not active against the metacestode stage of *E. multilocularis*, neither *in vivo* (Vanparijs, 1990), nor *in vitro,* as confirmed in this study. This could be explained by the fact that praziquantel causes paralysis of the parasite musculature, which then only affects actively moving, adult worms but not sessile metacestode larvae (Ritler et al., 2017; Vale et al., 2017). The antifungal agent amphotericin B (MMV689000) was shown to destroy *E. multilocularis* metacestodes *in vitro* at 2.7 μM (Reuter et al., 2003, 2010). Amphotericin B was also tested for treatment of human AE patients, but with limited success as the drug acted only parasitostatic and was accompanied with severe side effects (nephrotoxicity) (Reuter et al., 2003; Tappe et al., 2009). Amphotericin B was not active in our screen at 10 μM, as Reuter et al. (2003, 2010) employed a different cultivation system that required medium change (and consequently addition of new drugs each time) three times a week. Additionally, a different parasite strain and assay readout (assessing the numbers and sizes of vesicles) was employed. Another compound with known activity against *E. multilocularis* is nitazoxanide (MMV688991). It was previously shown to be active *in vitro* against *E. multilocularis* metacestodes at 3.3 μM (Stettler et al., 2003; Reuter et al., 2006), as well as against *E. granulosus* metacestodes and protoscoleces (Walker et al., 2004). Nitazoxanide was also tested *in vivo* in mice and in human patients suffering from CE or AE, but virtually no beneficial effects were observed (Pérez-Molina et al., 2011; Stettler et al., 2004; Tappe et al., 2009; Winning et al., 2009). Congruently, nitazoxanide was also among the 13 compounds from the Pathogen Box that were active at 10 μM in the present study, but it did not maintain its activity at 1 μM and was not further followed here. Mebendazole (MMV003152), together with ABZ, is the current standard chemotherapeutic treatment for AE patients. One of the first *in vitro* studies with *E. multilocularis* metacestodes demonstrated an inhibition of parasite proliferation over the course of three weeks treatment with mebendazole at 1 μM (Jura et al., 1998). Mebendazole was not active in our screen with a threshold of 20% relative activity compared to Tx-100, as the PGI-assay only identifies compounds that are active within a shorter time-span. This finding is line with our previous observations, where benzimidazoles only induced a slow release of PGI (Küster et al., 2014; Stadelmann et al., 2010). However, comparisons of benzimidazoles by electron microscopy showed that the drugs are having a clear effect on the metacestode ultrastructure early on (Küster et al., 2014). Auranofin (MMV688978) is a thioredoxin-glutathione reductase inhibitor that was shown to kill *E. granulosus* protoscoleces at 2.5 μM after 48 h (Bonilla et al., 2008). Consistent with these findings, the drug was also active against *E. multilocularis* metacestodes at 10 μM, but not at 1 μM. Mefloquine (MMV000016), originally developed and used against *Plasmodium*, has recently been found to be active against *E. multilocularis* both *in vitro*, as well as *in vivo* (Stadelmann et al., 2011; Küster et al., 2011, 2015; Rufener et al., 2018). Mefloquine has a rather high IC_50_ value against this parasite *in vitro* (>30 μM), but nevertheless it was identified in our screening at 10 μM. Taken together, the results of our present screening of the Pathogen Box correlate well with already known activities of specific drugs, underlining the power of the here employed screening cascade. Moreover, we identified four novel compounds with distinct *in vitro* activity against *E. multilocularis*.

So far, the Pathogen Box has been screened against the nematode *H. contortus* (Preston et al., 2016), the fungi *Candida albicans* and *Cryptococcus neoformans* (Vila and Lopez-Ribot, 2016; Mayer and Kronstad, 2017), *Plasmodium* and the kinetoplastids *Leishmania* and *Trypanosoma* (Calit et al., 2018; Dennis et al., 2018; Duffy et al., 2017), *Neospora caninum* (Müller et al., 2017)*, Mycobacterium abscessus* and *M. avium* (Jeong et al., 2018; Low et al., 2017), *Toxoplasma gondii* (Spalenka et al., 2017), *C. elegans* (Partridge et al., 2018), *Entamoeba histolytica* (Mi-Ichi et al., 2018), and *Giardia lamblia* and *Cryptosporidium parvum* (Hennessey et al., 2018). Interestingly, all compounds that exhibited activity against *E. multilocularis* were also active against at least one more pathogen other than the one it was selected for by MMV (with the exception of nitazoxanide), thus underlining the importance and potential of the concept of drug repurposing.

## 5 Conclusion

We identified two compounds (BPQ and MMV671636) within the 400 compounds of the MMV Pathogen Box with potent *in vitro* activities against *E. multilocularis* metacestodes. Moreover, we studied mitochondrial function in the parasite using a Seahorse XFp Analyzer and proved the cytochrome *bc*_1_ complex as a molecular target of BPQ in *E. multilocularis* GL cells. BPQ failed to be active *in vivo* in the murine model of AE. New, enhanced formulations of BPQ with increased bioavailability could overcome this problem in the future and hence lead to improved prognosis of patients suffering from echinococcosis. This study underlines that the repurposing of drugs has great potential when developing alternative treatment options against neglected diseases.

## Declarations of interest

None.

## References

[bib1] Agosin M. (1968). Biochemistry and physiology of echinococcus. Bull. World Health Organ..

[bib2] Birth D., Kao W.-C., Hunte C. (2014). Structural analysis of atovaquone-inhibited cytochrome *bc*_1_ complex reveals the molecular basis of antimalarial drug action. Nat. Commun..

[bib3] Bonilla M., Denicola A., Novoselov S.V., Turanov A.A., Protasio A., Izmendi D., Gladyshev V.N., Salinas G. (2008). Platyhelminth mitochondrial and cytosolic redox homeostasis is controlled by a single thioredoxin glutathione reductase and dependent on selenium and glutathione. J. Biol. Chem..

[bib4] Bryant C., Dawes B. (1970). Electron transport in parasitic helminths and Protozoa. Advances in Parasitology.

[bib5] Calit J., Dobrescu I., Gaitán X.A., Borges M.H., Ramos M.S., Eastman R.T., Bargieri D.Y. (2018). Screening the Pathogen Box against Plasmodium sexual stages using a new nanoluciferase based transgenic line of P. berghei identifies transmission-blocking compounds. Antimicrob. Agents Chemother..

[bib6] Conraths F.J., Deplazes P. (2015). Echinococcus multilocularis: epidemiology, surveillance and state-of-the-art diagnostics from a veterinary public health perspective.

[bib7] Croft S.L., Hogg J., Gutteridge W.E., Hudson A.T., Randall A.W. (1992). The activity of hydroxynaphthoquinones against Leishmania donovani. J. Antimicrob. Chemother..

[bib8] Dennis A.S.M., Rosling J.E.O., Lehane A.M., Kirk K. (2018). Diverse antimalarials from whole-cell phenotypic screens disrupt malaria parasite ion and volume homeostasis. Sci. Rep..

[bib9] Divakaruni A.S., Rogers G.W., Murphy A.N. (2014). Measuring mitochondrial function in permeabilized cells using the Seahorse XF analyzer or a clark-type oxygen electrode. Curr. Protoc. Toxicol..

[bib10] Duffy S., Sykes M.L., Jones A.J., Shelper T.B., Simpson M., Lang R., Poulsen S.-A., Sleebs B.E., Avery V.M. (2017). Screening the Medicines for malaria venture pathogen box across multiple pathogens reclassifies starting points for open-source drug discovery. Antimicrob. Agents Chemother..

[bib11] Garnier T., Mäntylä A., Järvinen T., Lawrence J., Brown M., Croft S. (2007). In vivo studies on the antileishmanial activity of buparvaquone and its prodrugs. J. Antimicrob. Chemother..

[bib12] Grüner B., Kern Petra, Mayer B., Gräter T., Hillenbrand A., Barth T.F.E., Muche R., Henne-Bruns D., Kratzer W., Kern Peter (2017). Comprehensive diagnosis and treatment of alveolar echinococcosis: a single-center, long-term observational study of 312 patients in Germany. GMS Infect. Dis..

[bib13] Hemphill A., Croft S.L. (1997). Electron microscopy in parasitology. Analytical Parasitology.

[bib14] Hemphill A., Spicher M., Stadelmann B., Mueller J., Naguleswaran A., Gottstein B., Walker M. (2007). Innovative chemotherapeutical treatment options for alveolar and cystic echinococcosis. Parasitology.

[bib15] Hemphill A., Stadelmann B., Rufener R., Spiliotis M., Boubaker G., Müller J., Müller N., Gorgas D., Gottstein B. (2014). Treatment of echinococcosis: albendazole and mebendazole – what else?. Parasite.

[bib16] Hennessey K.M., Rogiers I.C., Shih H.-W., Hulverson M.A., Choi R., McCloskey M.C., Whitman G.R., Barrett L.K., Merritt E.A., Paredez A.R., Ojo K.K. (2018). Screening of the Pathogen Box for inhibitors with dual efficacy against Giardia lamblia and Cryptosporidium parvum. PLoS Neglected Trop. Dis..

[bib17] Huang S.C.-C., Freitas T.C., Amiel E., Everts B., Pearce E.L., Lok J.B., Pearce E.J. (2012). Fatty acid oxidation is essential for egg production by the parasitic flatworm schistosoma mansoni. PLoS Pathog..

[bib18] Jeong J., Kim G., Moon C., Kim H.J., Kim T.H., Jang J. (2018). Pathogen Box screening for hit identification against Mycobacterium abscessus. PLoS One.

[bib19] Jura H., Bader A., Frosch M. (1998). In vitro activities of benzimidazoles against echinococcus multilocularis metacestodes. Antimicrob. Agents Chemother..

[bib20] Kammerer W.S., Perez-Esandi M.V. (1975). Chemotherapy of experimental Echinococcus granulosus infection. Trials in CF1 mice and jirds (Meriones unguiculatus). Am. J. Trop. Med. Hyg..

[bib21] Kern P. (2010). Clinical features and treatment of alveolar echinococcosis. Curr. Opin. Infect. Dis..

[bib22] Kern P., Menezes da Silva A., Akhan O., Müllhaupt B., Vizcaychipi K.A., Budke C., Vuitton D.A. (2017). The echinococcoses: diagnosis, clinical management and burden of disease. Adv. Parasitol..

[bib23] Küster T., Stadelmann B., Aeschbacher D., Hemphill A. (2014). Activities of fenbendazole in comparison with albendazole against Echinococcus multilocularis metacestodes in vitro and in a murine infection model. Int. J. Antimicrob. Agents.

[bib24] Küster T., Stadelmann B., Hermann C., Scholl S., Keiser J., Hemphill A. (2011). In vitro and in vivo efficacies of mefloquine-based treatment against alveolar echinococcosis. Antimicrob. Agents Chemother..

[bib25] Küster T., Stadelmann B., Rufener R., Risch C., Müller J., Hemphill A. (2015). Oral treatments of Echinococcus multilocularis-infected mice with the antimalarial drug mefloquine that potentially interacts with parasite ferritin and cystatin. Int. J. Antimicrob. Agents.

[bib26] Lawres L.A., Garg A., Kumar V., Bruzual I., Forquer I.P., Renard I., Virji A.Z., Boulard P., Rodriguez E.X., Allen A.J., Pou S., Wegmann K.W., Winter R.W., Nilsen A., Mao J., Preston D.A., Belperron A.A., Bockenstedt L.K., Hinrichs D.J., Riscoe M.K., Doggett J.S., Ben Mamoun C. (2016). Radical cure of experimental babesiosis in immunodeficient mice using a combination of an endochin-like quinolone and atovaquone. J. Exp. Med..

[bib27] Low J.L., Wu M.-L., Aziz D.B., Laleu B., Dick T. (2017). Screening of TB actives for activity against nontuberculous mycobacteria delivers high hit rates. Front. Microbiol..

[bib28] Luz A.L., Rooney J.P., Kubik L.L., Gonzalez C.P., Song D.H., Meyer J.N. (2015). Mitochondrial morphology and fundamental parameters of the mitochondrial respiratory chain are altered in Caenorhabditis elegans strains deficient in mitochondrial dynamics and homeostasis processes. PLoS One.

[bib29] Mäntylä A., Rautio J., Nevalainen T., Keski-Rahkonen P., Vepsälainen J., Järvinen T. (2004). Design, synthesis and in vitro evaluation of novel water-soluble prodrugs of buparvaquone. Eur. J. Pharmaceut. Sci..

[bib30] Marsolier J., Perichon M., DeBarry J.D., Villoutreix B.O., Chluba J., Lopez T., Garrido C., Zhou X.Z., Lu K.P., Fritsch L., Ait-Si-Ali S., Mhadhbi M., Medjkane S., Weitzman J.B. (2015). Theileria parasites secrete a prolyl isomerase to maintain host leukocyte transformation. Nature.

[bib31] Mayer F.L., Kronstad J.W. (2017). Discovery of a novel antifungal agent in the pathogen box. mSphere.

[bib32] McManus D.P., Smyth J.D. (1978). Differences in the chemical composition and carbohydrate metabolism of Echinococcus granulosus (horse and sheep strains) and E. multilocularis. Parasitology.

[bib33] Mi-Ichi F., Miyake Y., Tam V.K., Yoshida H. (2018). A flow cytometry method for dissecting the cell differentiation process of Entamoeba encystation. Front. Cell. Infect. Microbiol..

[bib34] Miyaji S., Katakura K., Matsufuji S., Murakami Y., Hayashi S., Oku Y., Okmoto M., Kamiya M. (1993). Failure of treatment with alpha-difluoromethylornithine against secondary multilocular echinococcosis in mice. Parasitol. Res..

[bib35] Müller J., Aguado A., Laleu B., Balmer V., Ritler D., Hemphill A. (2017). In vitro screening of the open source Pathogen Box identifies novel compounds with profound activities against Neospora caninum.

[bib36] Ortiz D., Forquer I., Boitz J., Soysa R., Elya C., Fulwiler A., Nilsen A., Polley T., Riscoe M.K., Ullman B., Landfear S.M. (2016). Targeting the cytochrome bc1 complex of Leishmania parasites for discovery of novel drugs. Antimicrob. Agents Chemother..

[bib37] Partridge F.A., Brown A.E., Buckingham S.D., Willis N.J., Wynne G.M., Forman R., Else K.J., Morrison A.A., Matthews J.B., Russell A.J., Lomas D.A., Sattelle D.B. (2018). An automated high-throughput system for phenotypic screening of chemical libraries on C. elegans and parasitic nematodes. Int. J. Parasitol. Drugs Drug Resist..

[bib38] Pérez-Molina J.A., Díaz-Menéndez M., Gallego J.I., Norman F., Monge-Maillo B., Ayala A.P., López-Vélez R. (2011). Evaluation of nitazoxanide for the treatment of disseminated cystic echinococcosis: report of five cases and literature review. Am. J. Trop. Med. Hyg..

[bib39] Preston S., Jiao Y., Jabbar A., McGee S.L., Laleu B., Willis P., Wells T.N.C., Gasser R.B. (2016). Screening of the ‘Pathogen Box’ identifies an approved pesticide with major anthelmintic activity against the barber's pole worm. Int. J. Parasitol. Drugs Drug Resist..

[bib40] Reuter S., Beisler T., Kern P. (2010). Combined albendazole and amphotericin B against Echinococcus multilocularis in vitro. Acta Trop..

[bib41] Reuter S., Buck A., Grebe O., Nüssle-Kügele K., Kern P., Manfras B.J. (2003). Salvage treatment with amphotericin B in progressive human alveolar echinococcosis. Antimicrob. Agents Chemother..

[bib42] Reuter S., Manfras B., Merkle M., Härter G., Kern P. (2006). In vitro activities of itraconazole, methiazole, and nitazoxanide versus echinococcus multilocularis larvae. Antimicrob. Agents Chemother..

[bib43] Ritler D., Rufener R., Sager H., Bouvier J., Hemphill A., Lundström-Stadelmann B. (2017). Development of a movement-based in vitro screening assay for the identification of new anti-cestodal compounds. PLoS Neglected Trop. Dis..

[bib44] Rufener R., Ritler D., Zielinski J., Dick L., da Silva E.T., da Silva Araujo A., Joekel D.E., Czock D., Goepfert C., Moraes A.M., de Souza M.V.N., Müller J., Mevissen M., Hemphill A., Lundström-Stadelmann B. (2018). Activity of mefloquine and mefloquine derivatives against Echinococcus multilocularis. Int. J. Parasitol. Drugs Drug Resist..

[bib45] Smith L., Serrano D.R., Mauger M., Bolás-Fernández F., Dea-Ayuela M.A., Lalatsa A. (2018). Orally bioavailable and effective buparvaquone lipid-based nanomedicines for visceral leishmaniasis. Mol. Pharm..

[bib46] Soni M.P., Shelkar N., Gaikwad R.V., Vanage G.R., Samad A., Devarajan P.V. (2014). Buparvaquone loaded solid lipid nanoparticles for targeted delivery in theleriosis. J. Pharm. BioAllied Sci..

[bib47] Spalenka J., Escotte-Binet S., Bakiri A., Hubert J., Renault J.-H., Velard F., Duchateau S., Aubert D., Huguenin A., Villena I. (2017). Discovery of new inhibitors of Toxoplasma gondii thanks to the Pathogen Box. Antimicrob. Agents Chemother..

[bib48] Spangenberg T., Burrows J.N., Kowalczyk P., McDonald S., Wells T.N.C., Willis P. (2013). The open access malaria box: a drug discovery catalyst for neglected diseases. PLoS One.

[bib49] Spiliotis M., Brehm K. (2009). Axenic in vitro cultivation of Echinococcus multilocularis metacestode vesicles and the generation of primary cell cultures. Methods Mol. Biol. Clifton NJ.

[bib50] Spiliotis M., Brehm K. (2004). Echinococcus multilocularis: identification and molecular characterization of a Ral-like small GTP-binding protein. Exp. Parasitol..

[bib51] Spiliotis M., Tappe D., Sesterhenn L., Brehm K. (2004). Long-term in vitro cultivation of Echinococcus multilocularis metacestodes under axenic conditions. Parasitol. Res..

[bib52] Stadelmann B., Küster T., Scholl S., Barna F., Kropf C., Keiser J., Boykin D.W., Stephens C.E., Hemphill A. (2011). In vitro efficacy of dicationic compounds and mefloquine enantiomers against Echinococcus multilocularis metacestodes. Antimicrob. Agents Chemother..

[bib53] Stadelmann B., Rufener R., Aeschbacher D., Spiliotis M., Gottstein B., Hemphill A. (2016). Screening of the open source malaria box reveals an early lead compound for the treatment of alveolar echinococcosis. PLoS Neglected Trop. Dis..

[bib54] Stadelmann B., Scholl S., Müller J., Hemphill A. (2010). Application of an in vitro drug screening assay based on the release of phosphoglucose isomerase to determine the structure–activity relationship of thiazolides against Echinococcus multilocularis metacestodes. J. Antimicrob. Chemother..

[bib55] Stettler M., Fink R., Walker M., Gottstein B., Geary T.G., Rossignol J.F., Hemphill A. (2003). In vitro parasiticidal effect of nitazoxanide against echinococcus multilocularis metacestodes. Antimicrob. Agents Chemother..

[bib56] Stettler M., Rossignol J.F., Fink R., Walker M., Gottstein B., Merli M., Theurillat R., Thormann W., Dricot E., Segers R., Hemphill A. (2004). Secondary and primary murine alveolar echinococcosis: combined albendazole/nitazoxanide chemotherapy exhibits profound anti-parasitic activity. Int. J. Parasitol..

[bib57] Tappe D., Müller A., Frosch M., Stich A. (2009). Limitations of amphotericin B and nitazoxanide in the treatment of alveolar echinococcosis. Ann. Trop. Med. Parasitol..

[bib58] Tsai I.J., Zarowiecki M., Holroyd N., Garciarrubio A., Sánchez-Flores A., Brooks K.L., Tracey A., Bobes R.J., Fragoso G., Sciutto E., Aslett M., Beasley H., Bennett H.M., Cai X., Camicia F., Clark R., Cucher M., De Silva N., Day T.A., Deplazes P., Estrada K., Fernández C., Holland P.W.H., Hou J., Hu S., Huckvale T., Hung S.S., Kamenetzky L., Keane J.A., Kiss F., Koziol U., Lambert O., Liu K., Luo X., Luo Y., Macchiaroli N., Nichol S., Paps J., Parkinson J., Pouchkina-Stantcheva N., Riddiford N., Rosenzvit M., Salinas G., Wasmuth J.D., Zamanian M., Zheng Y., Cai J., Soberón X., Olson P.D., Laclette J.P., Brehm K., Berriman M. (2013). The genomes of four tapeworm species reveal adaptations to parasitism. Nature.

[bib59] Vale N., Gouveia M.J., Rinaldi G., Brindley P.J., Gärtner F., Correia da Costa J.M. (2017). Praziquantel for schistosomiasis: single-drug metabolism revisited, mode of action, and resistance. Antimicrob. Agents Chemother..

[bib60] Vanparijs O. (1990). Chemotherapy of experimental*Echinococcus multilocularis* in jirds. Parasitol. Res..

[bib61] Vila T., Lopez-Ribot J.L. (2016). Screening the “pathogen box” for the identification of Candida albicans biofilm inhibitors. Antimicrob. Agents Chemother..

[bib62] Voorhis W.C.V., Adams J.H., Adelfio R., Ahyong V., Akabas M.H., Alano P., Alday A., Resto Y.A., Alsibaee A., Alzualde A., Andrews K.T., Avery S.V., Avery V.M., Ayong L., Baker M., Baker S., Mamoun C.B., Bhatia S., Bickle Q., Bounaadja L., Bowling T., Bosch J., Boucher L.E., Boyom F.F., Brea J., Brennan M., Burton A., Caffrey C.R., Camarda G., Carrasquilla M., Carter D., Cassera M.B., Cheng K.C.-C., Chindaudomsate W., Chubb A., Colon B.L., Colón-López D.D., Corbett Y., Crowther G.J., Cowan N., D'Alessandro S., Dang N.L., Delves M., DeRisi J.L., Du A.Y., Duffy S., El-Sayed S.A.E.-S., Ferdig M.T., Robledo J.A.F., Fidock D.A., Florent I., Fokou P.V.T., Galstian A., Gamo F.J., Gokool S., Gold B., Golub T., Goldgof G.M., Guha R., Guiguemde W.A., Gural N., Guy R.K., Hansen M.A.E., Hanson K.K., Hemphill A., Huijsduijnen R.H., van Horii T., Horrocks P., Hughes T.B., Huston C., Igarashi I., Ingram-Sieber K., Itoe M.A., Jadhav A., Jensen A.N., Jensen L.T., Jiang R.H.Y., Kaiser A., Keiser J., Ketas T., Kicka S., Kim S., Kirk K., Kumar V.P., Kyle D.E., Lafuente M.J., Landfear S., Lee N., Lee S., Lehane A.M., Li F., Little D., Liu L., Llinás M., Loza M.I., Lubar A., Lucantoni L., Lucet I., Maes L., Mancama D., Mansour N.R., March S., McGowan S., Vera I.M., Meister S., Mercer L., Mestres J., Mfopa A.N., Misra R.N., Moon S., Moore J.P., Costa F.M.R., da Müller J., Muriana A., Hewitt S.N., Nare B., Nathan C., Narraidoo N., Nawaratna S., Ojo K.K., Ortiz D., Panic G., Papadatos G., Parapini S., Patra K., Pham N., Prats S., Plouffe D.M., Poulsen S.-A., Pradhan A., Quevedo C., Quinn R.J., Rice C.A., Rizk M.A., Ruecker A., Onge R.S., Ferreira R.S., Samra J., Robinett N.G., Schlecht U., Schmitt M., Villela F.S., Silvestrini F., Sinden R., Smith D.A., Soldati T., Spitzmüller A., Stamm S.M., Sullivan D.J., Sullivan W., Suresh S., Suzuki B.M., Suzuki Y., Swamidass S.J., Taramelli D., Tchokouaha L.R.Y., Theron A., Thomas D., Tonissen K.F., Townson S., Tripathi A.K., Trofimov V., Udenze K.O., Ullah I., Vallieres C., Vigil E., Vinetz J.M., Vinh P.V., Vu H., Watanabe N., Weatherby K., White P.M., Wilks A.F., Winzeler E.A., Wojcik E., Wree M., Wu W., Yokoyama N., Zollo P.H.A., Abla N., Blasco B., Burrows J., Laleu B., Leroy D., Spangenberg T., Wells T., Willis P.A. (2016). Open source drug discovery with the malaria box compound collection for neglected diseases and beyond. PLoS Pathog..

[bib63] Walker M., Rossignol J.F., Torgerson P., Hemphill A. (2004). In vitro effects of nitazoxanide on Echinococcus granulosus protoscoleces and metacestodes. J. Antimicrob. Chemother..

[bib64] Wells T.N.C., Willis P., Burrows J.N., Hooft van Huijsduijnen R. (2016). Open data in drug discovery and development: lessons from malaria. Nat. Rev. Drug Discov..

[bib65] Williams D.L., Bonilla M., Gladyshev V.N., Salinas G. (2013). Thioredoxin glutathione reductase-dependent redox networks in platyhelminth parasites. Antioxidants Redox Signal..

[bib66] Winning A., Braslins P., McCarthy J.S. (2009). Case report: nitazoxanide for treatment of refractory bony hydatid disease. Am. J. Trop. Med. Hyg..

[bib67] Zaugg J.L., Lane V.M. (1992). Efficacy of buparvaquone as a therapeutic and clearing agent of Babesia equi of European origin in horses. Am. J. Vet. Res..

